# Rational Design of LiNi_1‐x_Fe_x_O_2_ Electrodes for Enhanced Oxygen Evolution Reaction Under Power‐Fluctuating Operations in Alkaline Water Electrolysis

**DOI:** 10.1002/smtd.70823

**Published:** 2026-07-08

**Authors:** Younghan Jung, Asiya M. Tamboli, Sowon Kim, Wan Sik Kim, Sathya Sheela Subramanian, Sejin Im, Junseok Sim, Junseok Oh, Geun Ho Gu, Chang‐Hee Kim

**Affiliations:** ^1^ School of Energy Technology Korea Institute of Energy Technology Jeonnam Republic of Korea

**Keywords:** alkaline water electrolysis, durability test, oxygen evolution reaction, power fluctuations, renewable energy

## Abstract

The electrochemical performance of alkaline water electrolyzers is hindered by power fluctuations when integrated with renewable energy sources in terms of electrode degradation. To address this, highly active and durable Fe‐incorporated LiNiO_2_ oxygen evolution reaction (OER) electrodes were fabricated for power‐fluctuating operations. The electrodes are synthesized through electrostatic spray deposition (ESD) as a one‐step, binder‐free, and scalable process. The electronic structure of LiNi_1‐_ *_x_* Fe*_x_* O_2_ was tuned by regulating the Ni and Fe content. This analysis suggests that optimal Fe incorporation raises the Ni oxidation state to Ni^3+^, which likely regulates the e_g_ orbital filling, thereby enhancing OER activity. Density functional theory (DFT) calculations corroborate this electronic modulation and further indicate that the Fe incorporation reduces the theoretical overpotential, consistent with experimental observations. The optimized electrocatalyst, LiNi_0.6_Fe_0.4_O_2_, exhibits remarkable OER performance with an overpotential (246 mV@10 mA cm^−2^) and a Tafel slope (41 mV dec^−1^). During half‐cell durability tests under various voltage cycling conditions, LiNi_0.6_Fe_0.4_O_2_ exhibits highly stable performance with insignificant degradation, attributed to stable maintenance of Ni^3+^. Moreover, the alkaline electrolyzer cell test achieved 87.7% voltage efficiency and stable operation for 200 h under power‐fluctuating conditions. These results highlight the potential of LiNi_0.6_Fe_0.4_O_2_ for application under variable renewable power supplies.

## Graphical abstract

We develop Fe‐incorporated LiNiO_2_ for the OER in alkaline media, where Fe enhances Ni^3^^+^ oxidation state and optimizes *e_g_* orbital filling, significantly improving activity under power fluctuations.

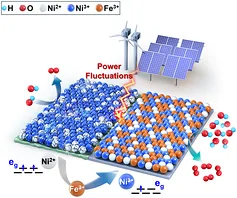

## 1 Introduction

It is evident that developing alternative energy sources is imperative due to the depletion of fossil fuels and their contribution to climate pollution, which results in a rise in global temperatures [1, 2]. Accordingly, water electrolysis coupled with renewable energy sources such as photovoltaic, wind power, and wave power has emerged as fascinating alternatives to solve this problem by converting energy into hydrogen without wasting renewable energy when there is no power demand [3, 4]. Moreover, compared to grid‐connected water electrolysis, hydrogen production using directly connected renewable energy sources can significantly reduce the carbon emissions for hydrogen production [5]. Therefore, to achieve true carbon neutrality, it is crucial to develop hydrogen production technologies that utilize water electrolysis directly linked to renewable energy. Currently, there is active research on water electrolysis, which consists of two main reactions: the hydrogen evolution reaction (HER) and the oxygen evolution reaction (OER). The OER is considered a more complex and sluggish reaction compared to the HER due to its 4‐electron transfer process, which implies that it requires higher energy to overcome the energy barrier [6]. Ir and Ru‐based materials have been considered the promising materials for the OER due to their high activity and stability in alkaline condition [7, 8]. However, these precious platinum‐group metals are gradually becoming scarce and expensive, making them difficult to utilize as the OER catalyst for large‐scale applications.

The use of precious metals as electrocatalysts for alkaline water electrolysis presents various challenges. Consequently, extensive research has focused on 3d transition metal‐based materials, which are non‐precious and highly active. These electrocatalysts include perovskite oxides [9], layered double hydroxides [10, 11], metal–organic frameworks [12], metal alkoxides [13], and high entropy oxides [14]. Among these materials, transition metal oxides are known to enhance the OER activity through electronic structural modification [15, 16, 17, 18, 19]. Several descriptors for electronic structural modification have been proposed. Suntivich et al. suggested that the binding energy of OH^−^ adsorption is optimized when the number of electrons in the e_g_ orbital of the 3d orbital of transition metals is close to 1.2 [9]. Additionally, some studies have estimated the hybridization between the 3d orbital of transition metals and the 2p orbital of oxygen through their covalency [17, 20]. According to these studies, stronger hybridization can enable faster injection and extraction of electrons between adsorbates and the transition metal surface, thereby facilitating charge transfer in the OER. These descriptors are controlled by the oxidation state of the transition metal. Therefore, some studies focus on modulating the oxidation state of Ni, which is a promising transition metal for the OER in alkaline water electrolysis [21, 22, 23, 24, 25]. It is reported that the enhanced degree of Ni 3d—O 2p hybridization, achieved by introducing Li into NiO, increases the oxidation state of Ni from 2+ to 3+. This increase in the oxidation state of Ni can facilitate fast kinetics for the OER [15]. Moreover, the number of electrons in the e_g_ orbital approaches 1.2 when the oxidation state of Ni from 2+ to 3+, optimizing the binding energy for the OER [9].

Fe is also a widely used material for the OER under alkaline media with Ni [18, 26]. Specifically, there are several reports on the synergistic effects between Ni and Fe [27], highlighting the following advantages: (1) Fe acts as an active site for OH^−^ to O‐O^−^ oxidation in a newly proposed OER mechanism for NiFe oxide [28, 29]. (2) Fe^3+^ induces the formation of a higher oxidation state of Ni by acting as a Lewis acid which is an electron acceptor [30]. (3) Fe prevents the undesired reaction of Ni, such as the corrosion, during the continuous OER in alkaline media [31].

As mentioned earlier, the electrolyzer must be integrated with renewable energy sources to produce green hydrogen for carbon neutrality. However, the renewable energy sources, such as photovoltaic, wind, and wave power, are fluctuating and intermittent, depending on location and weather conditions [32]. These power fluctuations have been a significant challenge for alkaline water electrolysis directly integrated with renewable energy. Sudden changes or shutdown in power can severely impact the electrode due to reverse current [33] or gas crossover [34], which can lead to reaching the explosion limit of hydrogen. When the reverse current flows through the electrode, oxidation occurs at the cathode, and reduction occurs at the anode for practical electrolyzer cell or stack. These contrary reactions are undesirable for each electrode, leading to degradation. Although several studies have addressed power fluctuation conditions [35, 36, 37], comprehensive research in this area remains limited. Therefore, it is essential to evaluate the stability of the electrode materials under power fluctuation conditions.

In this study, Fe was introduced into LiNiO_2_ in order to achieve a higher oxidation state of Ni and the synergistic effect between Ni and Fe. The OER performance of Fe‐introduced LiNiO_2_ was evaluated across a series of compositions and identified the optimal composition, LiNi_0.6_Fe_0.4_O_2_. Operational durability tests were conducted under various voltage cycling conditions for these two compositions, considering power‐fluctuating operations in a three‐electrode electrochemical system. To understand the degradation mechanism, various characterizations were carried out before and after these durability tests. Additionally, an alkaline electrolyzer cell durability test under fluctuating current conditions was evaluated using the large‐scale optimal composition sample to simulate practical use in renewable energy‐powered water electrolyzers. This study suggests a simple strategy to improve the OER activity by introducing Fe into LiNiO_2_. This incorporation increases the Ni^3+^ content, which optimizes the number of electrons in the e_g_ orbitals and strengthens the hybridization between Ni 3d and O 2p orbitals, while also utilizing the synergistic effect between Ni and Fe. Directly coupling water electrolyzers with renewable energy presents significant challenges due to power fluctuations. Therefore, this work is anticipated to provide valuable insights into degradation under such power‐fluctuating operations.

## 2 Experimental

### 2.1 Materials

Sandblasted circular nickel plate (SJ TECH, 20 mm in diameter) was used as the electrocatalyst substrate for the half‐cell OER test. Lithium chloride (LiCl, 99.95%, Sigma–Aldrich), nickel(II) chloride hexahydrate (NiCl_2_·6H_2_O, ReagentPlus, ≥98.5%, Sigma–Aldrich), iron(III) chloride (FeCl_3_, Reagent grade, 97%, Sigma–Aldrich), and ethyl alcohol (C_2_H_5_OH, ACS reagent, ≥99.5%, Sigma–Aldrich) were used as precursors and solvent. 1 m potassium hydroxide solution (KOH, Lab GRADE, DUKSAN PURE CHEMICALS) and 30 wt.% potassium hydroxide solution (KOH, Solution GRADE, DUKSAN PURE CHEMICALS), adjusted to pH 14, were used as the electrolyte for the half‐cell tests and alkaline electrolyzer cell tests, respectively. Foam‐type nickel metal was used as the electrocatalyst substrate of anode, cathode, and porous transport layer (PTL) for the alkaline electrolyzer cell test.

### 2.2 Precleaning of Ni Substrates

Before all electrocatalyst depositions, the circular nickel plate and nickel foam were cleaned using 1 m KOH, 20% hydrochloric acid solution (HCl, SAMCHUN), and deionized water (18.2 MΩ·cm at 25°C). Each step involved 5 min of cleaning in an ultrasonicator (POWERSONIC 510, HWASHIN). Afterward, the substrates were dried using inert nitrogen gas (N_2_, INDUSTRIAL 99.9% grade, DAEDEOK GAS., LTD.).

### 2.3 Sample Preparation

For LiNiO_2_ preparation, 0.1 m LiCl solution and 0.1 m NiCl_2_·6H_2_O solution in ethyl alcohol, were mixed to form the precursor solution. For LiNi_0.8_Fe_0.2_O_2_, 0.1 m LiCl solution, 0.08 m NiCl_2_·6H_2_O solution, and 0.02 m FeCl_3_ solution, dissolved in ethyl alcohol, were combined. Other compositions were prepared similarly, using the corresponding concentrations of each precursor. Each precursor solution was stirred gently using a Digital Hotplate Stirrer (MSH‐20D, DAIHAN Scientific) to ensure uniform blending. The prepared solution was transferred into a syringe, fitted with a metal nozzle. The solution in the syringe was sprayed onto the nickel plate substrate via an electrostatic spray machine (ESR200R2, NanoNC) at a rate of 40 µL min^−1^ for 3.6 mL at 70°C, with a voltage of 20 kV applied between the metal nozzle and the substrate stage, where the substrate was placed. The deposited samples were then heated on a Digital Hotplate Stirrer at 290°C for 1 h in atmospheric conditions to remove impurities and promote oxidation. For nickel foam used in the alkaline electrolyzer cell tests, only the deposition volume was increased to 9 mL, while all other deposition parameters are the same. The actual catalyst loadings of the optimized composition on the Ni plate and the Ni foam were 0.43 and 0.67 mg cm^−2^, respectively. Considering the difference in their double‐layer capacitance (C_dl_) values (Ni plate: 5.08 mF cm^−2^ and Ni foam: 8.26 mF cm^−2^), the catalyst mass per effective reaction area remained highly consistent between the two different setups.

### 2.4 Characterizations

General surface morphologies were observed by scanning electron microscopy (SEM; Helios G5 UX, Thermo Fisher Scientific) at 10 kV and 0.1 nA, equipped with a secondary electron detector.

High resolution transmission electron microscopy (HRTEM) and High‐angle annular dark‐field scanning transmission electron microscopy (HAADF‐STEM) images, and STEM‐Energy dispersive X‐ray spectroscopy (EDS) elemental mapping were performed at 300 kV with a double aberration‐corrected STEM system (Spectra Ultra, Thermo Fisher Scientific, NFEC‐2023‐01‐284572) equipped with Ultra‐X 6‐EDS detectors. The STEM, STEM‐EDS elemental mapping Images and spectra were processed using the Velox software from Thermo Scientific. Drift correction of specimen was conducted during the acquisition. Each elemental map is constructed by integrating the signal from La‐Lα, Sr‐Lα, Ni‐Kα, Ti‐Kα and O‐Kα characteristic X‐rays, respectively. The EDS maps were processed by the average filter to minimize random noise, which do not contain artifact from filtering. For TEM analysis, the as‐prepared LiNiO_2_ and LiNi_0.6_Fe_0.4_O_2_ were soaked in ethanol, and the deposited layers were detached using a sonicator for 1 h. Approximately 5 to 10 droplets of the sample‐ethanol mixture were dropped onto a Lacey carbon film 200 mesh Cu grid (Electron Microscopy Science).

X‐ray Photoelectron Spectroscopy (XPS; NEXSA, Thermo Fisher Scientific) was performed to investigate the electronic structure and ionic ratio for samples using an Al Kα (1486.6 eV) X‐ray source. The Ni and Fe 2p spectra were fitted with Gaussian‐Lorentzian peaks (30% Gaussian) under a Shirley‐type background, and the fitting, as well as the calculation of binding energies and peak areas, was performed using the XPSPEAK41 software.

X‐ray diffractometer (SmartLab, Rigaku) was used to investigate X‐ray diffraction (XRD) analysis for crystallinity. X‐ray power is 9 kW due to observing thin film samples and copper source was used. To compare the crystallinity of LiNiO_2_ through XRD, the crystalline samples were prepared using the same deposition process, followed by heat treatments at 500°C and 600°C.

### 2.5 Electrochemical Measurements

Electrochemical measurements, such as linear sweep voltammetry (LSV), cyclic voltammetry (CV), electrochemical impedance spectroscopy (EIS), chronopotentiometry (CP), and chronoamperometry (CA) were performed in a customized three‐electrode configuration connected to a potentiostat (SP‐240, BioLogic). The three‐electrode configuration was comprised of a customized working electrode with the prepared samples, a graphite counter electrode, and a Hg/HgO reference electrode (ALS Co., Ltd, RE‐61AP) in a 1 m KOH. The potentials measured by the Hg/HgO reference electrode were converted into the potentials versus reversible hydrogen electrode by following calculation, E(V vs. RHE) = E(V vs. Hg/HgO) + 0.0977 + 0.05916 × pH, and which were represented as vs. RHE. Additionally, the calibrated potentials versus RHE were corrected by eliminating the IR drop to remove the effect of solution resistance (R_s_). The R_s_ was measured by electrochemical impedance spectroscopy (EIS) under current density of 10 mA cm^−2^, ranging from 100 kHz to 100 mHz. The EIS data were fitted using EC‐Lab software (Bio‐Logic) based on an equivalent circuit model (Figure S1). In this model, R1 represents the R_s_, and R2 corresponds to the charge transfer resistance (R_ct_) related to the OER kinetics at the electrode‐electrolyte interface. Q2, constant phase element (CPE) was employed instead of a pure capacitor to account for the non‐ideal capacitive behavior arising from the electrode surface. The high reliability of the fits was confirmed by the normalized error (χ^2^/|Z|^2^) values for all samples, which were consistently below 10^−2^.

All the samples were activated using CV with 10 cycles and a scan rate of 5 mV s^−1^. Subsequently, the OER performance was evaluated as LSV curves with a scan rate of 5 mV s^−1^. The LSV curves were utilized for identifying the overpotential at a current density of 10 mA cm^−2^ for each sample and constructing the Tafel plot in overpotential versus logarithmic current density. The overpotentials (*η*) for OER were calculated by subtracting the standard water oxidation potential, as shown below:

$$\eta =E_{RHE}-1.23V$$

Tafel plots were derived from the LSV curves over a current density range of 25–100 mA cm^−2^. The derived Tafel equation is as follows:

η=a+blogj

where *η* is the overpotential, *a* is Tafel constant related to exchange current density, *b* is Tafel slope, and *j* is the current density.

Additionally, the CV was conducted to measure the electrochemical surface area (ECSA) to evaluate the active area of the samples across a series of scan rates: 5, 10, 15, 20, and 25 mV s^−1^, in the non‐Faradaic region (Figure S2). The ECSA was calculated using the following equation:

$$ECSA=\frac{C_{dl}}{C_{s}}$$

where C*_dl_* is the double‐layer capacitance measured from the CV curves, and C*_s_* is the specific capacitance, typically taken as 0.04 mF cm^−2^ in 1 m KOH electrolyte [38].

CA was conducted for operational durability tests. Before this technique, a potentiodynamic polarization curve was derived from LSV at 2 mV sec^−1^ of scan rate. According to the diagram, CA was performed in three regimes as follows: constantly applied voltage of 0.9 V (vs. Hg/HgO) for Regime 1. For Regime 2, alternatively applied 0.9 and 0.5 V (vs. Hg/HgO) at 1 min intervals, and for Regime 3, alternatively applied 0.9 and 0.3 V (vs. Hg/HgO) at 1 min intervals. Each regime was maintained for 23 h (Figure 6b). The reproducibility of initial current density at applied voltage of 0.9 V is performed with ten independent experiments to represent the error range (Figure S3). Error bars and average current density for initial current densities, represented by the maximum and minimum range from at least ten independent measurements of each composition.

### 2.6 Alkaline Electrolyzer Cell Test

Nickel foam with an area of 21.12 cm^2^ was used as the anode, substrate, and PTL for both the cathodic and anodic compartments in the alkaline electrolyzer cell configuration. The optimized anode was prepared using the LiNi_0.6_Fe_0.4_O_2_ precursor, which was deposited through electrostatic spraying with a total spray volume of 9 mL. The cell assemblies consisted of the prepared LiNi_0.6_Fe_0.4_O_2_ or Ni foam anode, a commercial Zirfon diaphragm (Agfa, Zirfon UTP 220) as the separator, and a Raney Ni [39, 40, 41] as the cathode. The Raney Ni was fabricated by sputtering an Al target onto Ni foam, followed by heat treatment to form an intermetallic compound. The Al was then leached using a solution of 30 wt.% KOH and 10 wt.% KNaC_4_H_4_O_6_·4H_2_O to form a porous Ni structure. The components were assembled with PTL under a pressure of 400 kgf.

The alkaline electrolyzer cell test was conducted using a custom‐manufactured alkaline water electrolyzer and a potentiostat (SP‐150e, BioLogic) equipped with a booster (FlexP0012, BioLogic). 30 wt.% KOH solution was used as the electrolyte, flowing at a rate of 500 sccm at 80°C. Currents densities were applied to the cell in 14 sequences, ranging from 0.008 to 0.8 A cm^−2^, and the corresponding cell voltages were measured before and after the durability test. From these measurements, *I*–*V* curves were plotted, showing cell voltage as a function of current density, with currents normalized by the electrode area of 21.12 cm^2^. Voltage efficiency was calculated as follows:

$$Voltage\;efficiency=\frac{Thermoneutral\;voltage\left(V_{th},1.48V\right)}{Measured\;cell\;voltage}\times 100$$

The durability test was performed using the CP technique, alternating between open‐circuit voltage (OCV) and 0.4 A cm^−2^ every 5 min, for a total duration of 200 h.

### 2.7 Computational Details

DFT calculations were performed using the Vienna ab initio Simulation Package [42] within the generalized gradient approximation using the Perdew–Burke–Ernzerhof [43] functional. Core‐valence interactions were described using projector‐augmented wave method [44] potentials with a plane‐wave cutoff of 520 eV. Convergence criteria were set to 10^−5^ eV for total energy and 0.05 eV Å^−1^ for forces. Strong correlation effects of Ni and Fe 3d states were treated using the Hubbard *U* approach (*U*_eff_  =  to 6.40 eV for Ni and 5.30 eV for Fe) [45]. Brillouin zone sampling employed a Γ‐centered 5 × 3 × 1 Monkhorst–Pack grid [46].

The XRD consistent LiNiO_2_ structure (MP ID: mp‐25411) was used as the starting point. Slab models were constructed along the (110) and (104) plane with a vacuum spacing of 15 Å; the bottom two layers were fixed during relaxation. LiNi_0.6_Fe_0.4_O_2_ was modeled by substituting 5 out of 12 transition metal sites with Fe atoms.

The OER was analyzed using the standard four‐electron mechanisms,

(1) $$H_{2}O\left(l\right)+\;*\;\to OH*\;+H^{+}+e^{-}$$

(2) $$OH*\;\to O*\;+H^{+}+e^{-}$$

(3) $$H_{2}O\left(l\right)+O*\;+\to OOH*\;+H^{+}+e^{-}$$

(4) $$OOH*\;\to O_{2}\left(g\right)+\;*\;+H^{+}+e^{-}$$

where ^*^ denotes a catalytic site. The overall reaction is [47]

(5) $$2H_{2\;}O_{\left(l\right)}+*\;\to \;O_{2\;\left(g\right)}+4H^{+}+4e^{-}\;\Delta G_{0}=4.92\;eV$$

Free energies were calculated using the computational hydrogen electrode framework [48] at standard conditions (pH 0, T = 298.15 K, U = 0 V). The relative free energy of intermediates was computed as:

(6) $$\Delta G_{OH*}=E_{OH*}^{DFT}-\;E_{*}^{DFT}-\;\mu_{H2O\left(l\right)}^{o}+\frac{1}{2}\;\mu_{H2\left(g\right)}^{o}+{\hat{G}}_{OH*}$$

(7) $$\Delta G_{O*}=E_{O*}^{DFT}-\;E_{*}^{DFT}-\;\mu_{H2O\left(l\right)}^{o}+\mu_{H2\left(g\right)}^{o}+{\hat{G}}_{O*}$$

(8) $$\Delta G_{OOH*}=E_{OOH*}^{DFT}-\;E_{*}^{DFT}-\;2\mu_{H2O\left(l\right)}^{o}+\frac{3}{2}\;\mu_{H2\left(g\right)}^{o}+{\hat{G}}_{OOH*\;}$$

where $E_{i}^{DFT}$ denotes total energies of species *i* while $\mu_{H2O(l)}^{o}$ and $\mu_{H2(g)}^{o}$ are the chemical potentials of liquid water (approximated as the H_2_O_(g)_ at vapor pressure) and hydrogen gas, respectively. Vibrational contribution correction of species *i*, ${\hat{G}}_{i\;}$, of 0.35, 0.05 and 0.40 eV were applied for OH^*^, O^*^ and OOH^*^, respectively. The calculated energies are tabulated in Table S3.

The reaction energies for each step were defined as:

(9) $$\Delta \;G_{1}=\Delta G_{OH*}$$

(10) $$\Delta \;G_{2}=\Delta G_{O*}\;-\Delta G_{OH*}$$

(11) $$\Delta \;G_{3}=\Delta G_{OOH*}\;-\Delta G_{O*}$$

(12) $$\Delta \;G_{4}=\Delta G_{0}\;-\Delta G_{OOH*}$$

The potential‐determining step was identified as the maximum free‐energy change:

(13) $$G^{OER}=\;\max\;\left[\Delta G_{1},\;\Delta G_{2},\;\Delta G_{3},\;\Delta G_{4},\right]$$

and the theoretical overpotential was calculated as:

(14) $$\eta^{OER}=\;\left(G^{OER}\;/e\right)-1.23\;V$$

## 3 Results and Discussion

### 3.1 Optimization of Heating Treatment Temperature

Prior to characterizing the materials and evaluating OER performance for Fe‐incorporated compositions, the crystallinity of LiNiO_2_ was assessed under various heat treatment temperatures. Through this preliminary study, the optimal heating temperature for sample preparation was confirmed. Then the electrochemical performance of these samples was evaluated to determine the optimal heat treatment temperature. As described in the methods section of this study, the samples were prepared by heating at 290°C to eliminate impurities after the deposition process. To achieve crystalline LiNiO_2_, further heat treatments were conducted at relatively higher temperatures of 500°C and 600°C using a furnace. The XRD patterns, shown in Figure S4 a, confirm the crystallinity of the samples subjected to higher heat treatments. These patterns align with the JCPDS card for LiNiO_2_ (Figure S5, JCPDS card no. 00‐009‐0063), with peaks corresponding to specific 2θ values. In contrast, the sample heated at 290°C primarily shows strong Ni metal peaks, which originate from the substrate used for deposition. This result indicates that LiNiO_2_ prepared at 290°C is amorphous or polycrystalline and lacks crystallinity. Electrochemical measurements, presented in Figure S4b–d, were conducted for comparison. The non‐crystalline LiNiO_2_ demonstrates superior OER performance compared to the crystalline LiNiO_2_. Notably, the sample heated at 600°C exhibits the lowest OER performance among all tested samples. Based on these results, it is clear that LiNiO_2_ forms a highly crystalline phase under high‐temperature conditions. This leads to lower electrical conductivity and poorer electrochemical performance. Therefore, all compositions samples were prepared at optimal heating temperature of 290°C.

### 3.2 Characterization of Surface Morphology and Crystallinity

**FIGURE 1:**
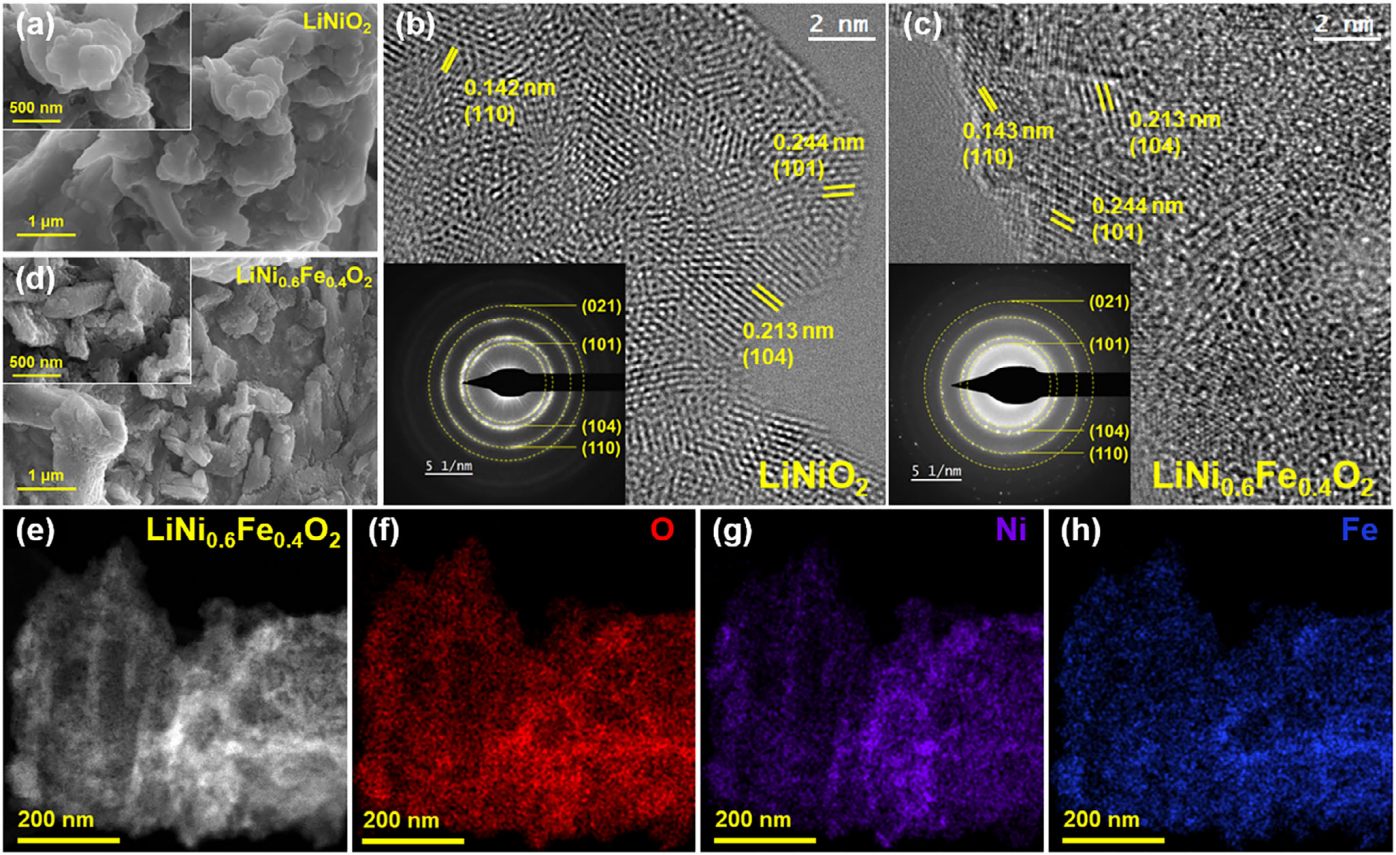
SEM surface images in 1 µm scale (inset: 500 nm scale) for (a) LiNiO_2_ and (d) LiNi_0.6_Fe_0.4_O_2_. HRTEM images in 2 nm scale (inset: SAED pattern) for (b) LiNiO_2_ and (c) LiNi_0.6_Fe_0.4_O_2_. (e) HAADF‐STEM image and EDS mapping images of (f) O, (g) Ni, and (h) Fe for LiNi_0.6_Fe_0.4_O_2_.

The surface morphology of pure LiNiO_2_ in Figure 1a, optimized LiNi_0.6_Fe_0.4_O_2_ in Figure 1d, and other Fe substituted samples in Figure S6 were observed using scanning electron microscopy (SEM). Compared to LiNi_0.6_Fe_0.4_O_2_, some large particles, likely resulting from agglomeration, were observed in LiNiO_2_. Additionally, the surface of LiNi_0.6_Fe_0.4_O_2_ appeared rougher than that of LiNiO_2_. Although LiNi_0.6_Fe_0.4_O_2_ seems to have a larger surface area than LiNiO_2_, the precise reactive area for the electrochemical reaction is discussed in the electrochemical measurements section. High‐resolution transmission electron microscopy (HRTEM) images of LiNiO_2_ and LiNi_0.6_Fe_0.4_O_2_ in Figure 1b,c revealed partial crystallinity, indicating that the samples are polycrystalline. These images showcase the lattice fringes corresponding to specific lattice planes. The corresponding diffraction patterns are also shown in the selected area electron diffraction (SAED) patterns (inset of Figure 1b,c), which exhibit characteristic rings and random spots. The lattice planes and diffraction patterns of LiNiO_2_ and LiNi_0.6_Fe_0.4_O_2_ were identified through HRTEM analysis. Specifically, this was achieved by matching the lattice distances measured from the fringes with the reference XRD pattern of LiNiO_2_ (Figure S5, JCPDS card no. 00‐009‐0063). Both compositions include the lattice fringes of 0.142 or 0.143, 0.213, and 0.244 nm, corresponding to the (110), (104), and (101) planes, respectively. These HRTEM images comparison implies that there is no structural change with Fe incorporation. Elemental mapping was conducted using energy‐dispersive spectroscopy (EDS) with high‐angle annular dark‐field scanning transmission electron microscopy (HAADF‐STEM) for LiNi_0.6_Fe_0.4_O_2_, as shown in Figure 1e–h. The mapping analysis revealed that Ni and Fe are uniformly distributed in the oxide state, with a measured atomic ratio of Ni to Fe of 22.00: 14.28, approximately 6: 4 (Table S1). This confirms that the Ni‐to‐Fe ratio in the sample aligns with the composition used during sample preparation.

### 3.3 Characterization of Electronic Structures

**FIGURE 2:**
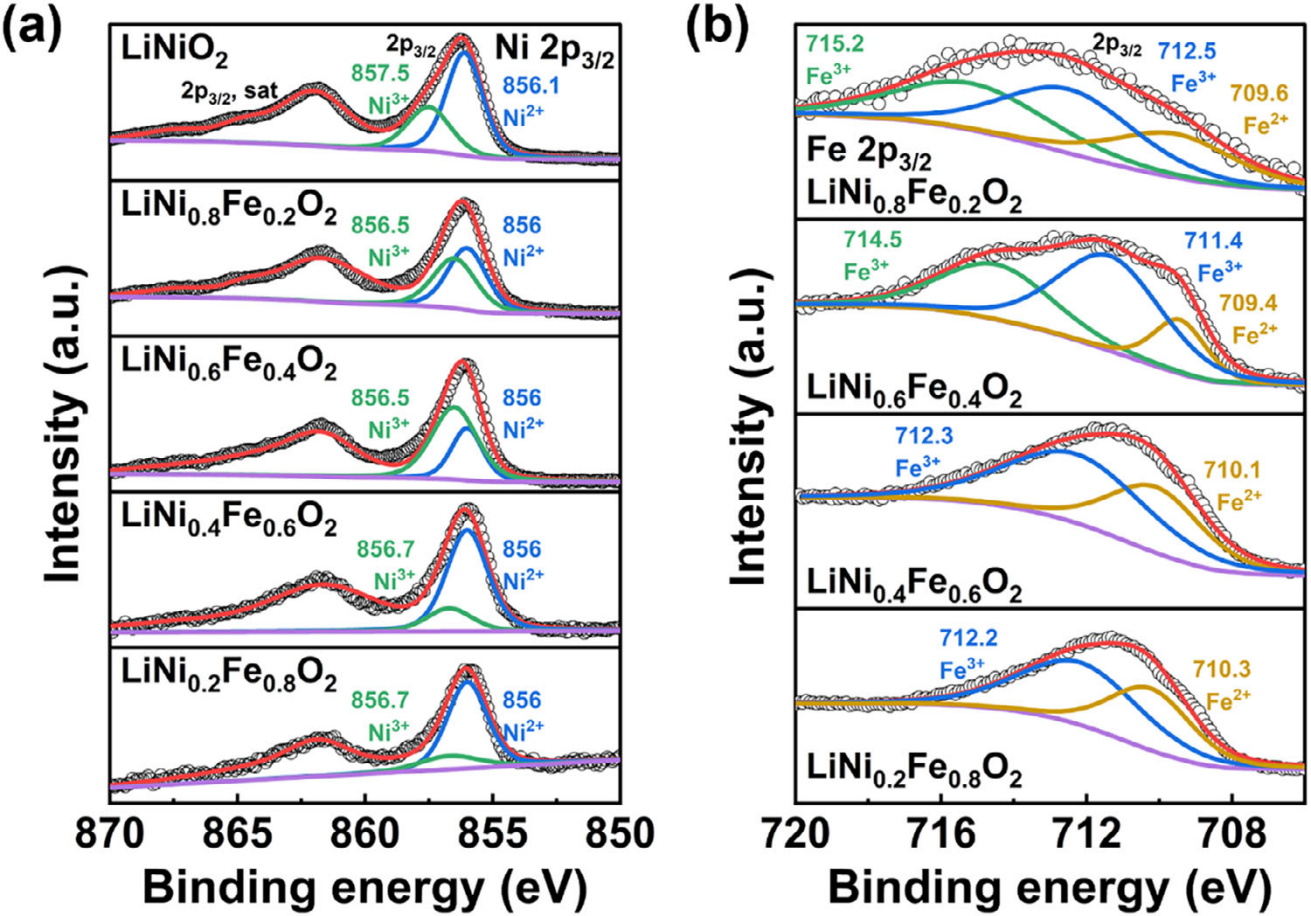
XPS spectra of (a) Ni 2p_3/2_ and (b) Fe 2p_3/2_ for LiNi_1‐_ *_x_* Fe*_x_* O_2_, with *x* = 0, 0.2, 0.4, 0.6, 0.8, respectively (The raw data, Shirley baseline, and fitted envelope are presented as hollow dots, a purple line, and a red line, respectively).

To verify the electronic structure, X‐ray photoelectron spectroscopy (XPS) analysis was conducted Figure 2 shows the deconvoluted Ni 2p_3/2_ and Fe 2p_3/2_ XPS spectra for LiNi_1‐_ *_x_* Fe*_x_* O_2_ (*x* = 0.2, 0.4, 0.6, 0.8). The Ni 2p_3/2_ spectra are shown in Figure 2a, highlighting peak shifts due to Fe introduction. The oxidation states of Ni are modified by changing the chemical composition through this introduction. In the case of LiNiO_2_ (*x* = 0), the Ni^2+^ peak appears at a binding energy of 856.1 eV, and the Ni^3+^ peak at 857.5 eV [49, 50]. In this composition, the relative peak areas of Ni^2+^ and Ni^3+^ are 64.7% and 35.3% respectively (Table S2). For LiNi_0.8_Fe_0.2_O_2_ (*x* = 0.2), the first Fe‐incorporated composition, the Ni^3+^ peak shifts to 856.5 eV [49, 50], while the Ni^2+^ peak position remains unchanged. This shift implies that the electronic structure is modified through Fe incorporation. Additionally, the Ni^3+^ peak area increases to 47.3%. Subsequently, in LiNi_0.6_Fe_0.4_O_2_ (*x* = 0.4), the Ni^3+^ peak position remains the same as in LiNi_0.8_Fe_0.2_O_2_. However, its area increases significantly to 67.1%, representing an approximately 32% increase compared to LiNiO_2_. For LiNi_0.4_Fe_0.6_O_2_ (*x* = 0.6) and LiNi_0.2_Fe_0.8_O_2_ (*x* = 0.8), no further shift in the Ni^3+^ peak position is observed; nevertheless, the peak areas decrease significantly compared to other compositions. This trend suggests that there is an optimal Fe substitution point, rather than a direct proportional relationship between Fe incorporation and Ni^3+^ content. The optimized composition exhibits the highest Ni^3+^ content, indicating that the number of the e_g_ orbital is closer to 1.2, along with stronger hybridization between the 3d and 2p orbitals compared to Ni^2+^‐dominant compositions [17, 20]. Based on this analysis, it is expected that the optimized composition demonstrates enhanced OER activity and charge transfer.

The Fe 2p_3/2_ spectra shown in Figure 2b exhibited differences depending on the ratio of Fe substitution. In LiNi_0.8_Fe_0.2_O_2_ and LiNi_0.6_Fe_0.4_O_2_, the Fe^3+^ peaks were split into 712.5 and 715.2 eV for LiNi_0.8_Fe_0.2_O_2_, and 711.4 and 714.5 eV for LiNi_0.6_Fe_0.4_O_2_ [51, 52, 53]. Notably, these two compositions exhibited Fe^3+^ peaks at relatively higher binding energies, which are consistent with the Fe^3+^ peaks of FeOOH. In contrast, Fe^3+^ peaks at relatively lower binding energies are indicative of Fe_2_O_3_. A higher binding energy indicates that an atom in a molecule tends to withdraw electrons from other atoms, acting as an electron acceptor. Therefore, the Fe^3+^ peaks with higher binding energies in these compositions tend to withdraw electrons from Ni, promoting the formation of Ni^3+^. This behavior contrasts with other compositions that do not exhibit these higher binding energy peaks. As mentioned earlier in the Ni 2p_3/2_ spectra shown in Figure 2a, LiNi_0.8_Fe_0.2_O_2_ and LiNi_0.6_Fe_0.4_O_2_ exhibited a higher Ni^3+^ ratio compared to LiNi_0.4_Fe_0.6_O_2_ and LiNi_0.2_Fe_0.8_O_2_. The trend observed in the Ni spectra supports this hypothesis. On the other hand, for LiNi_0.4_Fe_0.6_O_2_ and LiNi_0.2_Fe_0.8_O_2_, the Fe spectra exhibited only two peaks corresponding to Fe^2+^ and Fe^3+^. The Fe^3+^ peaks appeared at 712.3 and 712.2 eV [51, 52, 53] for LiNi_0.4_Fe_0.6_O_2_ and LiNi_0.2_Fe_0.8_O_2_, respectively, which have similar peak positions to the lower binding energy peaks of LiNi_0.8_Fe_0.2_O_2_ and LiNi_0.6_Fe_0.4_O_2_. This indicates that the absence of the higher binding energy Fe^3+^ peaks is responsible for the lower Ni^3+^ ratio in these two compositions.

### 3.4 Electrochemical Measurements

**FIGURE 3:**
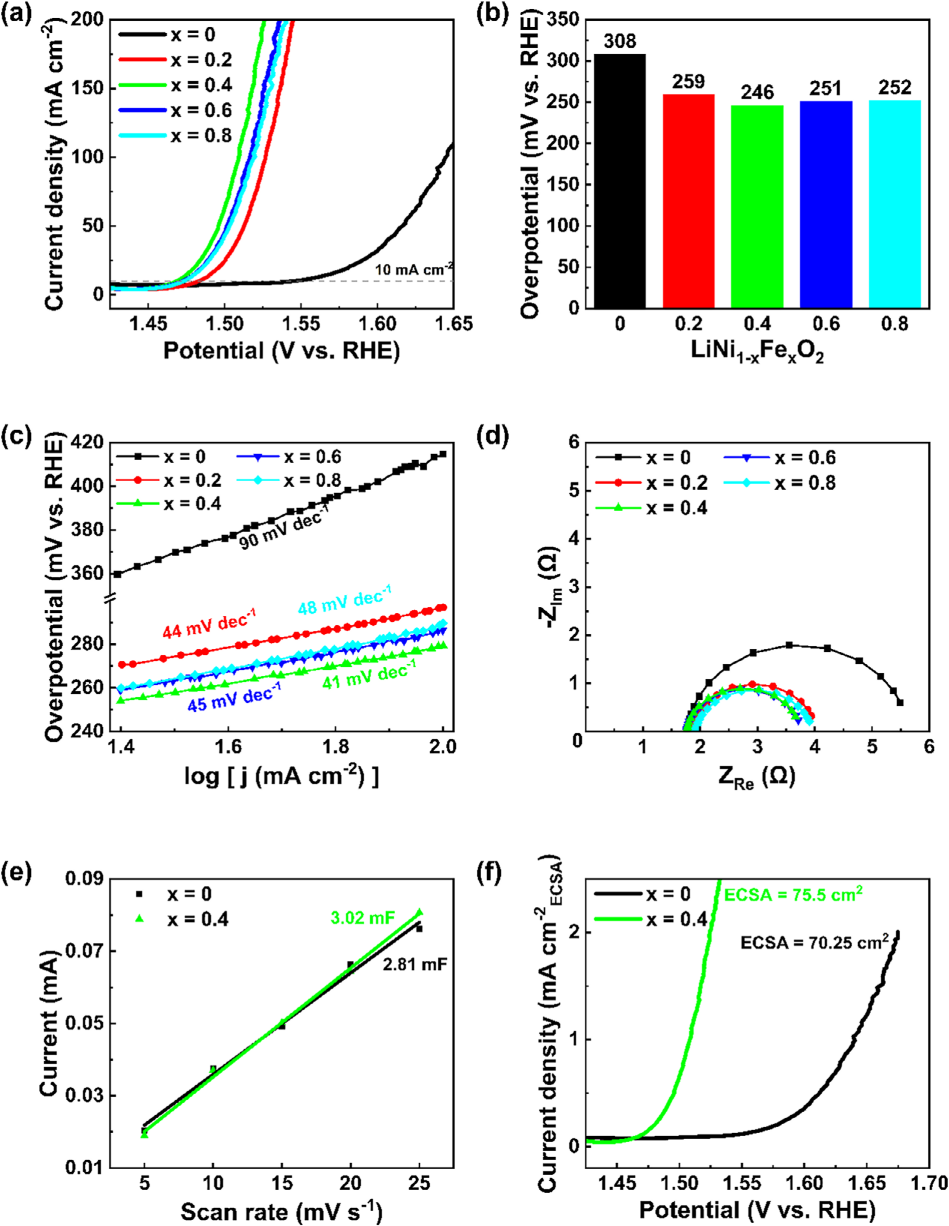
(a) Linear sweep voltammetry (LSV) curves, (b) overpotentials at a current density of 10 mA cm^−2^, (c) Tafel plots, and (d) electrochemical impedance spectroscopy (EIS) Nyquist plots for LiNi_1‐_ *_x_* Fe*_x_* O_2_ with *x* = 0, 0.2, 0.4, 0.6, and 0.8. (e) Estimated double‐layer capacitance (C*_dl_*) values of LiNi_1‐_ *_x_* Fe*_x_* O_2_ with *x* = 0, and 0.4 and (f) ECSA normalized LSV curves for LiNiO_2_ and LiNi_0.6_Fe_0.4_O_2_. The overpotential values in (b) represent the average of four independent measurements of each composition.

Electrochemical performance measurements were conducted for pure LiNiO_2_ and Fe‐incorporated samples, where Ni was substituted with Fe at increments of 0.2 m in solution. According to the LSV graph in Figure 3a, the Fe‐incorporated samples exhibit better performance than pure LiNiO_2_ (represented in *x* = 0 in Figure 3) suggesting that the synergistic effect between Ni and Fe. Notably, LiNi_0.6_Fe_0.4_O_2_ (represented as *x* = 0.4 in Figure 3), with a composition ratio Ni: Fe = 6: 4, demonstrates the best performance. However, the OER performance decreased after this composition. A similar trend in overpotential was observed at a current density of 10 mA cm^−2^ in Figure 3b. Pure LiNiO_2_ requires 308 mV of potential to achieve this current density, whereas the best‐performing composition, LiNi_0.6_Fe_0.4_O_2_, requires only 246 mV which is 62 mV lower than that of pure LiNiO_2_. This indicates that LiNi_0.6_Fe_0.4_O_2_ reaches the desired potential more efficiently, implying superior activity. These results suggest that as the e_g_ orbital filling approaches 1.2, adsorption between adsorbate and the electrocatalyst is optimized by increasing the oxidation state from Ni^2+^ to Ni^3+^. As shown in Figure 3c, Tafel plots were plotted from the LSV curves for current densities ranging from 25 to 100 mA cm^−2^ (1.4 ∼ 2.0 in the log scale), where the OER actively occurs. According to these plots, LiNi_0.6_Fe_0.4_O_2_ exhibits the lowest Tafel slope of 41 mV dec^−1^. This low value indicates that current density increases sharply with a smaller change in overpotential, suggesting superior charge transfer kinetics in the OER compared to other compositions. EIS measurements in Figure 3d were conducted at a current density of 10 mA cm^−2^ to evaluate the solution resistance and charge transfer resistance. In these plots, LiNi_0.6_Fe_0.4_O_2_ exhibits the fastest electron transfer between the adsorbate and catalyst surface, with the smallest charge transfer resistance of 1.985 Ω, whereas the pure LiNiO_2_ shows nearly twice this at 3.856 Ω. The decrease in Tafel slope and charge transfer resistance are attributed to stronger hybridization, which results from the increased oxidation state of Ni due to Fe incorporation. SEM images indicate that the LiNi_0.6_Fe_0.4_O_2_ has more roughness and porosity than the LiNiO_2_. However, it does not represent the actual reactive area for the OER. To estimate the precise area, electrochemical surface area (ECSA) is calculated using cyclic voltammetry (CV) technique. This allows us to determine whether enhanced OER performance is due to the modification of electronic structure or an increase in reactive surface area. The C*_dl_* is measured as the slope between the charging current and scan rate in Figure 3e. The ECSA is calculated by dividing C*_dl_* by the C*_s_* [38], yielding values of 70.25 cm^2^ for LiNiO_2_ and 75.5 cm^−2^ for LiNi_0.6_Fe_0.4_O_2_. Normalizing the LSV curves with both the geometrical surface area and ECSA in Figure 3f reveals the same trend between the normalized LSV curves, indicating that the effect of surface area is negligible. Therefore, we conclude that the electric structure modifications contribute more significantly to the improvement of the OER performance.

### 3.5 Elucidation of the Active Sites With DFT Calculations

**FIGURE 4:**
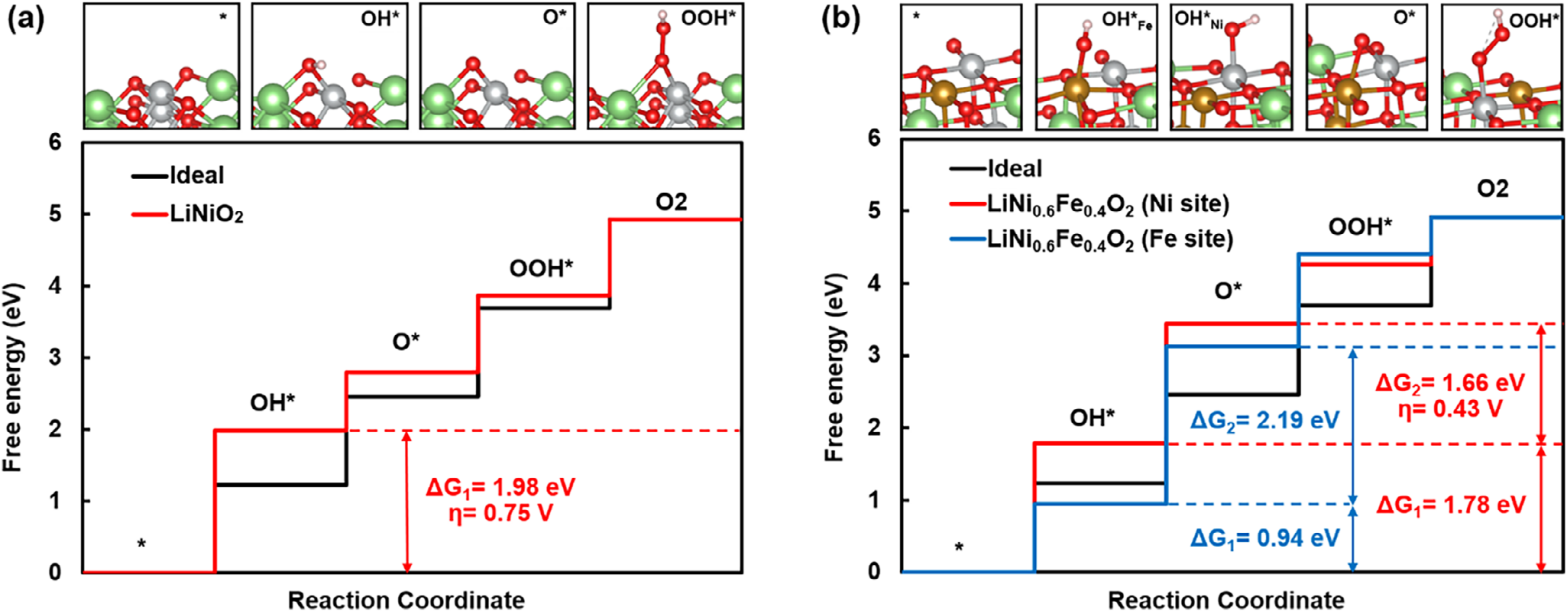
Free energy diagrams for OER intermediates on (a) LiNiO_2_ and (b) LiNi_0.6_Fe_0.4_O_2_. The results indicate that the potential‐determining step is changed and overpotential is reduced upon Fe incorporation on LiNiO_2_. The top inset images show relaxed structures for ^*^, OH^*^, O^*^ and OOH^*^ species, respectively. Brown, gray, green, red, and white spheres are Fe, Ni, Li, O, and H atoms, respectively.

Density functional theory (DFT) calculations were performed to elucidate the four‐electron OER mechanism on LiNiO_2_ and LiNi_0.6_Fe_0.4_O_2_ to identify the active sites and the potential‐determining step (PDS). As shown in Figure 4a for LiNiO_2_, the OH^*^ adsorption is the PDS with Gibbs free energy of 1.98 eV, corresponding to an overpotential of 0.75 V. Upon Fe incorporation, a surface slab model with neighboring Ni and Fe sites was used to unveil the synergistic OER mechanism (Figure 4b). The presence of multiple active site combinations enables more segmented reaction pathways. Notably, the reaction free energy for the initial OH^*^ adsorption step significantly decreases from 1.98 to 0.94 eV at the Fe site. While subsequent step to O^*^ on the Fe site is energetically demanding at 2.19 eV, the transition of the adsorbed OH^*^ to an adjacent Ni site is thermodynamically spontaneous at the equilibrium potential of 1.23 V. Following this transition, it is more favorable for Ni to act as the active site, as it requires a lower free energy. These suggest a synergistic catalytic mechanism. The Fe sites are highly favorable for the initial OH^*^ adsorption step, while the Ni sites preferentially drive the subsequent intermediate steps in the OER mechanism. Consequently, it is evident that Fe incorporation exerts a highly positive catalytic effect from a mechanistic perspective.

**FIGURE 5:**
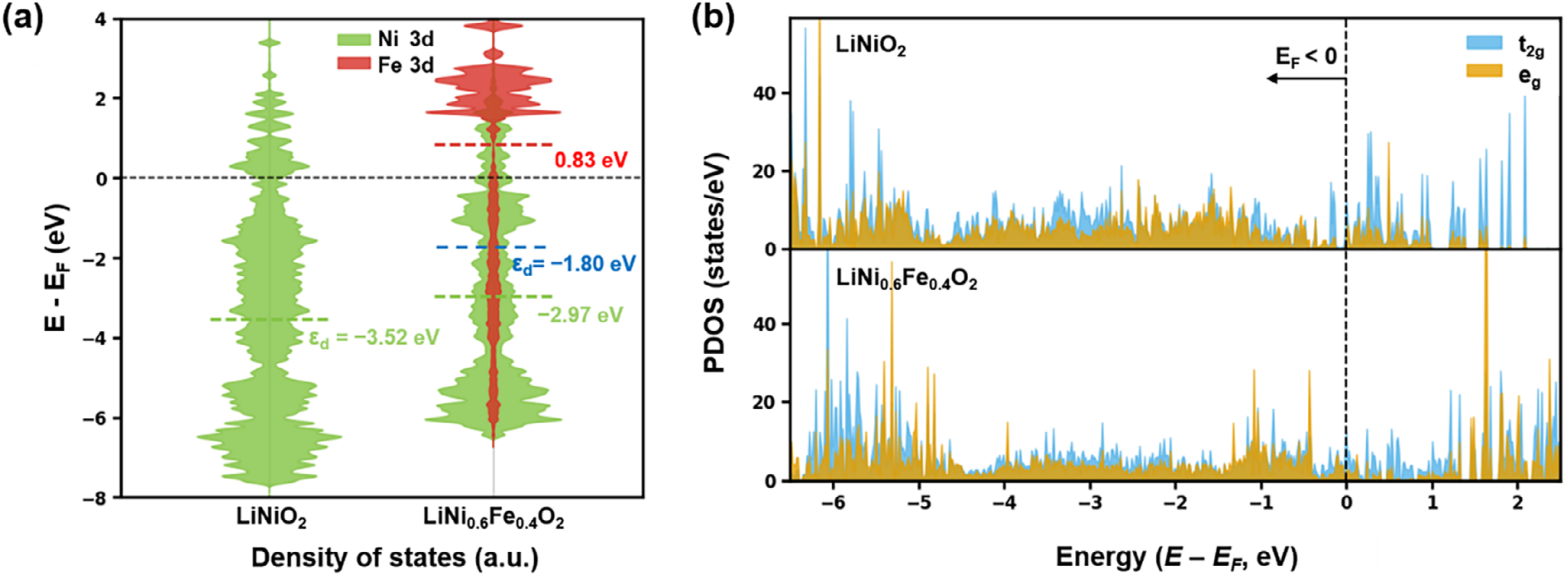
The d‐orbital Projected Density of states (PDOS) analysis for each catalyst. (a) Symmetrical plot of total d‐orbital along the vertical axis, with dotted lines indicating the d‐band centers of each metal species. The blue dotted line indicates the total d‐band center of the LiNi_0.6_Fe_0.4_O_2_ catalyst. (b) PDOS plot for split d‐orbital occupancy. All energies are referenced to the Fermi level (E_F_).

The position of the d‐band center affects the adsorption energy of OER intermediates [54, 55]. To gain insights into the OER activity and the role of Fe incorporation, the Projected Density of states (PDOS) was obtained. The PDOS energy scales were referenced to the Fermi levels (E_F_), which were calculated as −1.85 and −3.25 eV for LiNiO_2_ and LiNi_0.6_Fe_0.4_O_2_, respectively. As shown in Figure 5a, electronic structure of LiNi_0.6_Fe_0.4_O_2_ is characterized by the presence of Fe 3d states positioned at higher energies relative to the Ni 3d states. Specifically, the strong hybridization between Fe 3d and O 2p orbitals pushes the anti‐bonding states above the E_F_. This distribution of high‐energy Fe states shifts the overall d‐band center (*ε*_d_) upward from −3.52 to −1.80 eV, bringing it closer to the E_F_. To provide a detailed electronic analysis, the d‐orbital states in Figure 5b are resolved into e_g_ and t_2g_ sub‐orbitals. Quantitative analysis reveals that, upon Fe incorporation, the integrated e_g_ occupancy below the E_F_ was decreased to 67% relative to that of pristine LiNiO_2_. Such a depletion of e_g_ electrons and the positive shift of the *ε*_d_ are consistent with enhanced adsorption, leading to the optimized binding with OER intermediates. Relaxed slab images and PDOS distributions of each element are provided in Figure S7.

### 3.6 Operational Durability Test

**FIGURE 6:**
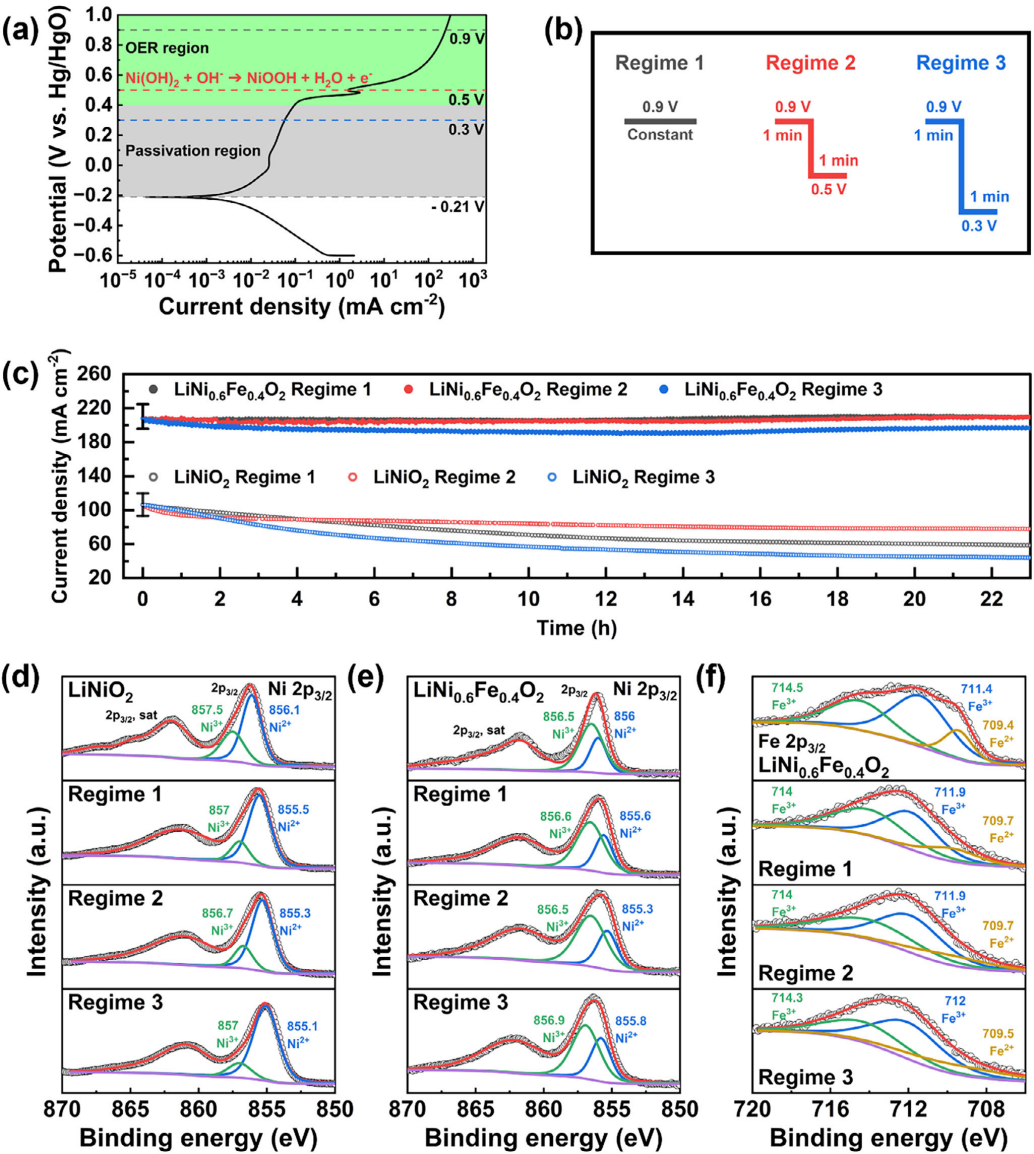
(a) Potentiodynamic polarization curve of LiNi_0.6_Fe_0.4_O_2_ for operational durability test. (b) Schematic of operational durability test with corresponding regime. (c) Operational durability test results normalized to the initial average current density for LiNi_0.6_Fe_0.4_O_2_ and LiNiO_2_ under Regimes 1, 2 and 3, measured by the chronoamperometry (CA) technique. Comparison of Ni 2p_3/2_ XPS spectra for (d) LiNiO_2_ and (e) LiNi_0.6_Fe_0.4_O_2_, and (f) Fe 2p_3/2_ XPS spectra for LiNi_0.6_Fe_0.4_O_2_ before and after each regime (The raw data, Shirley baseline, and fitted envelope are presented as hollow dots, a purple line, and a red line, respectively).

As mentioned earlier, power fluctuation has been a crucial obstacle to producing green hydrogen via alkaline water electrolysis integrated with renewable energy. Therefore, it is essential to develop electrodes with enhanced stability against power fluctuations. In this study, the durability of LiNiO_2_ and LiNi_0.6_Fe_0.4_O_2_ was evaluated using 3‐electrode half‐cell tests under specific voltage cycling protocols. First, using the potentiodynamic polarization curve (Figure 6a), we identified the OER region (On‐state) and the passivation (Off‐state) region. Based on these observations, the applied voltage for the durability test was divided into three distinct regimes (Figure 6b) to reflect real‐world renewable energy profiles. Regime 1 involved the application of a constant high voltage (0.9 V vs Hg/HgO) in the OER region. This represents an operating condition where the electrolyzer receives a stable and sufficient supply of renewable energy (e.g., constant solar and wind power). For Regime 2, the voltage was applied within the OER region (between 0.9 and 0.5 V vs Hg/HgO). This introduces the electrolyzer to a fluctuating load within the On‐state, simulating periods when the renewable energy supply varies but the electrolyzer does not completely turn off (e.g., variations in daytime solar irradiance or inconsistent wind power). Furthermore, Regime 3 introduced fluctuations by dropping the voltage down to the Off‐state (0.9 to 0.3 V vs Hg/HgO) to evaluate the electrolyzer under complete On/Off cycling conditions. This reflects real‐world scenarios where the renewable energy supply completely stops and restarts (e.g., solar power at night or intermittent wind conditions). In the case of LiNiO_2_ (Figure 6c), the current density decreases by 44.88% in Regime 1. On the other hand, LiNi_0.6_Fe_0.4_O_2_ exhibits stable performance with minimal degradation during the testing period. The same trend is observed for Regime 2, while the current density of LiNiO_2_ decreases by 26.56%. The highest degradation rates for each composition are observed in Regime 3, the most variable condition. LiNiO_2_ degrades by more than half, 58.39%, while LiNi_0.6_Fe_0.4_O_2_ shows only a 5.17% decrease in performance. This degradation trend is expected due to the harsh voltage cycling in Regime 3, which includes on and off states.

To assess the degradation of samples after the durability test, XPS analysis was conducted for each composition before and after the test. From the spectra for LiNiO_2_ in Figure 6d, the peak areas of Ni^3+^ are severely decreased by 15.3%, 12.3%, and 20.4% after Regime 1, 2, and 3, respectively. The Ni^3+^ peak shifts are also severer than optimized ones from 857.5 to 857, 856.7, and 857 eV after Regime 1, 2, and 3, respectively. From the spectra in Figure 6e, in contrast, the peak areas of Ni^3+^ decreased by 0.7%, 0.8%, and 4.0% after Regime 1, 2, and 3, respectively for LiNi_0.6_Fe_0.4_O_2_. Additionally, the Ni^3+^ shows slight peak shifts from 856.5 to 856.6 eV after Regime 1, to 856.5 eV after Regime 2, and to 856.9 eV after Regime 3 (Table S4). In the Fe 2p XPS spectra after the durability test regimes shown in Figure 6f, the Fe^2+^ and Fe^3+^ peaks generally decreased. Furthermore, the inductively coupled plasma optical emission spectroscopy (ICP‐OES) analysis (Table S5) reveals that while the Fe concentration in the electrolytes after Regimes 1 and 2 showed no noticeable difference compared to the pristine electrolyte, an increased Fe concentration was clearly detected after Regime 3. Taken together, these results imply that the XPS signal changes in Regimes 1 and 2 primarily reflect electronic structural modifications, whereas the repeated on and off states in Regime 3 eventually induces Fe dissolution, which correlates with the observed durability degradation. This suggests that Fe participated in side reactions instead of Ni, likely acting as a protective mechanism to prevent Ni from undergoing side reactions, such as Ni corrosion, during the continuous OER process [31, 56]. Further experiments will be conducted to clarify these results. The minimal changes in Ni^3+^ peak areas for LiNi_0.6_Fe_0.4_O_2_ suggest that Ni^3+^ ions are well maintained, with only slight peak shifts, indicating chemically more stable compared to LiNiO_2_. These finding correlate with the more stable current densities measured for LiNi_0.6_Fe_0.4_O_2_ compared to LiNiO_2_. As shown in Figure S8, electrochemical performance metrics such as overpotential, Tafel slope, and charge transfer resistance were also influenced by Ni^3+^ peak area. In the case of LiNi_0.6_Fe_0.4_O_2_, the descriptors remained stable, whereas LiNiO_2_ were significantly unstable with changes in the peak area.

The surface morphology changes after the durability test were observed through SEM images for both LiNiO_2_ and LiNi_0.6_Fe_0.4_O_2_. As shown in the images of Figure S9a–d for LiNi_0.6_Fe_0.4_O_2_ before and after Regime 1, 2, and 3, no significant agglomeration or morphological changes are observed, with the porous and rough surfaces remaining well‐maintained. In contrast, the SEM images for LiNiO_2_ shown in Figure S9e–h reveals that the surfaces become considerably smoother after Regime 1 and 2. Additionally, after Regime 3, the particles on surface appear more agglomerated than those in LiNi_0.6_Fe_0.4_O_2_. Although we conclude that the surface area does not contribute to the OER performance, the significant surface degradation observed in LiNiO_2_ is substantial enough to impact the performance.

While these durability results in the half‐cell configuration provide promising initial insights, evaluating the long‐term durability under realistic operational conditions is essential to fully validate its practical applicability. Therefore, we further extended our evaluation to a comprehensive 200‐h long‐term durability test in an alkaline electrolyzer cell (full‐cell) configuration, as discussed in the subsequent section.

### 3.7 Alkaline Electrolyzer Cell Durability Test

**FIGURE 7:**
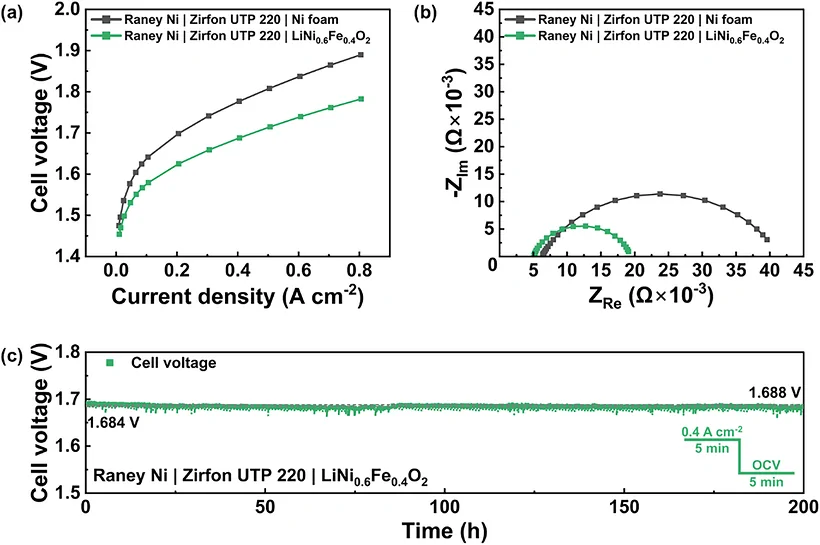
Comparison of (a) *I*–*V* curves and (b) EIS Nyquist plots for LiNi_0.6_Fe_0.4_O_2_ and nickel foam electrode configurations. (c) Alkaline electrolyzer cell durability test result for LiNi_0.6_Fe_0.4_O_2_ anode under fluctuating current operations, alternating between 0.4 A cm^−2^ and OCV for 5 min over 200 h (inset: schematic of the alkaline electrolyzer cell durability test).

Based on these half‐cell results, we further evaluated the optimized large‐scale electrode (21.12 cm^2^) in an alkaline water electrolyzer cell to reflect the operating conditions of actual commercial electrolyzers. Since the half‐cell tests identified the On/Off cycling in Regime 3 as the most severe condition, we adopted this On/Off protocol for the electrolyzer cell durability evaluation. Furthermore, to closely reflect the actual power control of industrial operations during the fluctuating conditions, we regulated the On/Off cycles of the electrolyzer cell using current control rather than voltage control. To simulate practical commercial operations, the alkaline electrolyzer test apparatus is designed with Balance of Plant (BOP) components to be comparable to commercial electrolyzers. These assessments are essential to verify performance stability when an alkaline water electrolyzer is directly coupled with renewable energy sources. The alkaline electrolyzer cell configuration consists of the as‐prepared LiNi_0.6_Fe_0.4_O_2_ or nickel foam as the anode, Raney Ni as the cathode, and Zirfon UTP 220 as the separator. The alkaline electrolyzer cell test included electrochemical measurements, such as *I*–*V* curves and EIS Nyquist plots, as well as a durability test at 0.4 A cm^−2^ and OCV conditions for 200 h. *I*–*V* curves and EIS Nyquist plots for LiNi_0.6_Fe_0.4_O_2_ anode were plotted before and after the durability test, as presented in Figure S10. The detailed sequence of *I*–*V* curves is explained in the methods section. In the comparison of *I*–*V* curves between LiNi_0.6_Fe_0.4_O_2_ and the nickel foam anode, as shown in Figure 7a, the nickel foam anode exhibited a higher cell voltage than LiNi_0.6_Fe_0.4_O_2_ across the entire current density range. Notably, LiNi_0.6_Fe_0.4_O_2_ demonstrated a superior cell voltage efficiency of 87.7% at a current density of 0.4 A cm^−2^, compared to 83.3% for the nickel foam anode. In the EIS Nyquist plots shown in Figure 7b, the lower charge transfer resistance of 0.014 Ω is attributed to the high cell voltage efficiency of LiNi_0.6_Fe_0.4_O_2_. As shown in Figure 7c, the cell voltage increased slightly from 1.684 to 1.688 V under fluctuating current operation over the 200 h durability test, corresponding to an increase of approximately 0.24% and a low degradation rate of 0.02 mV h^−1^. This result demonstrates the stable maintenance of the cell voltage during the operation. Additionally, as shown by the *I*–*V* curves in Figure S10a, the cell voltage at a current density of 0.4 A cm^−2^ exhibited minimal shift from 1.688 to 1.684 V. Despite being tested at large scale and under power‐fluctuated operations, LiNi_0.6_Fe_0.4_O_2_ exhibited highly stable performance, indicating its potential for practical applications.

## 4 Conclusion

In this study, the optimized composition, LiNi_0.6_Fe_0.4_O_2_, exhibited significant OER performance with an overpotential of 246 mV, a Tafel slope of 41 mV dec^−1^, and a charge transfer resistance of 1.985 Ω. This composition showed the highest Ni^3+^ ratio (67.07%) among the LiNi_1‐_ *_x_* Fe*_x_* O_2_ compositions, compared to LiNiO_2_ (35.33%). These results suggest that enhanced OER performance is closely associated with the increased oxidation state of Ni^3+^. Furthermore, DFT calculations theoretically corroborated this, indicating that a synergistic mechanism between Ni and Fe optimizes the intermediate adsorption energies through e_g_ orbital modulation and a positive d‐band center shift. During operational durability tests, LiNi_0.6_Fe_0.4_O_2_ maintained stable performance under constant voltage in the OER region and voltage cycling within the OER region. In addition, it exhibited a negligible current density decrease of 5% under fluctuations down to the off state, in comparison to LiNiO_2_ which showed 58% decrease. Notably, the Ni^3+^ content in LiNi_0.6_Fe_0.4_O_2_ was well maintained after various operational durability tests as compared to LiNiO_2_. Finally, the alkaline electrolyzer cell test was conducted on a large‐scale anode for practical applications under current‐fluctuating operations. LiNi_0.6_Fe_0.4_O_2_ anode exhibited cell voltage efficiency of 87.7% at a current density of 0.4 A cm^−2^. Moreover, this electrode demonstrated stable durability with only a 0.24% performance drop and a low degradation rate of 0.02 mV h^−1^ over 200 h under on‐off operation. This work proposes a simplified approach to enhancing the OER performance and provides valuable insights into the rational design of industrially viable OER electrode for renewable energy powered alkaline water electrolyzers.

## Conflicts of Interest

The authors declare no conflicts of interest.

## Supporting information




**Supporting File**: smtd70823‐sup‐0001‐SuppMat.docx.

## Data Availability

The data that support the findings of this study are available from the corresponding author upon reasonable request.
