# Supplementary material for: Rational Design of LiNi1‐xFexO2 Electrodes for Enhanced Oxygen Evolution Reaction Under Power‐Fluctuating Operations in Alkaline Water Electrolysis
Source: Small Methods. 2026 Jul 8;10(18):e70823. doi: 10.1002/smtd.70823 (PMC13599704; doi:10.1002/smtd.70823)
Supplement: Supplementary file 1 [file SMTD-10-e70823-s001.docx]

Supporting Information

**Rational Design of LiNi_1-x_Fe_x_O_2_ Electrodes for Enhanced Oxygen Evolution Reaction under Power-fluctuating Operations in Alkaline Water Electrolysis**

*Younghan Jung, Asiya M. Tamboli, Sowon Kim, Wan Sik Kim, Sathya Sheela Subramanian, Sejin Im, Junseok Sim, Junseok Oh, Geun Ho Gu, and Chang-Hee Kim*^*^

Y. Jung, A. M. Tamboli, S. Kim, W. S. Kim, S. S. Subramanian, S. Im, J. Sim, J. Oh, G. H. Gu, C. Kim

School of Energy Technology, Korea Institute of Energy Technology, Jeonnam 58330, Republic of Korea
E-mail: chkim@kentech.ac.kr

**Figure S1.** Equivalent circuit model used for electrochemical impedance spectroscopy (EIS) fitting. R1, R2, and Q2 represent the solution resistance (R_s_), charge transfer resistance (R_ct_), and constant phase element (CPE), respectively.

**Figure S2.** Double-layer capacitance measurements for determining ECSA with CV measured in a non-Faradaic region at scan rates of 5, 10, 15, 20, and 25 mV s^-1^ for (a) LiNiO_2_ and (b) LiNi_0.6_Fe_0.4_O_2_.

**Figure S3.** Error bars with average current density for initial current densities represented by the maximum and minimum range from at least ten independent measurements of each composition.

**Figure S4.** (a) XRD patterns, (b) LSV curves, (c) overpotentials at a current density of 10 mA cm^-2^, and (d) EIS plots for LiNiO_2_ at various heating temperatures.

**Figure S5.** Reference LiNiO_2_ XRD pattern from JCPDS card no. 00-009-0063.

**Figure S6.** SEM surface images in 1 μm scale (inset: 500 nm scale) for (a) LiNi_0.8_Fe_0.2_O_2_, (b) LiNi_0.4_Fe_0.6_O_2_, and (c) LiNi_0.2_Fe_0.8_O_2_.

**Figure S7.** Electronic structure analysis via spin-polarized density functional theory calculations. (a) Perspective views of the relaxed (110) slab structures for LiNiO_2_ and (b) LiNi_0.6_Fe_0.4_O_2_, visualized using vesta^[1]^. (c) Projected Density of states (PDOS) profiles for LiNiO_2_ and (d) LiNi_0.6_Fe_0.4_O_2_ of each element.

**Figure S8.** Changes in electrochemical performance metrics before and after operational durability test, correlated with the Ni^3+^ peak area.

**Figure S9.** SEM surface images in 1 μm scale (inset: 500 nm scale) for (a-d) LiNi_0.6_Fe_0.4_O_2_ and (e-h) LiNiO_2_ before and after regime 1, 2, and 3.

**Figure S10.** (a) I-V curves and (b) EIS Nyquist plots for LiNi_0.6_Fe_0.4_O_2_ before and after 200 h durability test.

**Figure S11.** LSV curves for (a) LiNi_0.6_Fe_0.4_O_2_ and (b) LiNiO_2_ before and after operational durability test with regime 1, 2 and 3.

**Figure S12.** Error bars with average overpotentials represented by standard deviation (s.d.) from four independent measurements of each composition.

**Figure S13.** Operational durability test results normalized to the initial average current density for LiNi_0.6_Fe_0.4_O_2_ and LiNiO_2_ under regimes 1, 2 and 3, measured by the chronoamperometry (CA) technique over 23 hours.

**Figure S14.** Comparison of the oxygen evolution reaction (OER) performance with reported various transition metal oxides.

**Figure S15.** Photographs of (a) the alkaline electrolyzer cell configuration and (b) its components.

**Figure S16.** Mott-Schottky plots of the Fe-incorporated LiNi_1-_*_x_*Fe*_x_*O_2_ (*x* = 0.2, 0.4, 0.6, and 0.8) samples, measured in the non-Faradaic region.

**Table S1.** EDS atomic ratio for LiNi_0.6_Fe_0.4_O_2_

| Element | Atomic ratio [%] |
| --- | --- |
| Ni | 22.00 |
| Fe | 14.28 |
| O | 63.71 |

| Element | Atomic ratio [%] |
| --- | --- |
| Ni | 22.04 |
| Fe | 14.39 |
| O | 63.56 |

**Table S2.** XPS spectra peak ratios for LiNiO_2_ and LiNi_0.6_Fe_0.4_O_2_ (Average areas ± standard deviation derived from three independent fitting iterations)

|  | Ni^2+^ Area [%] | Ni^3+^ Area [%] |
| --- | --- | --- |
| LiNiO_2_ | 64.7 (±1.2) | 35.3 (±1.2) |
| LiNi_0.6_Fe_0.4_O_2_ | 32.9 (±0.3) | 67.1 (±0.3) |

**Table S3.** Calculated free energies of OER intermediates (OH*, O* and OOH*) for the LiNiO_2_ and LiNi_0.6_Fe_0.4_O_2_

|  | LiNiO_2_ | LiNi_0.6_Fe_0.4_O_2_ (Fe) | LiNi_0.6_Fe_0.4_O_2_ (Ni) |
| --- | --- | --- | --- |
| ΔG_OH*_ (eV) | 1.98 | 0.94 | 1.78 |
| ΔG_O*_ (eV) | 2.80 | 3.13 | 3.44 |
| ΔG_OOH*_ (eV) | 3.86 | 4.40 | 4.25 |

**Table S4.** XPS spectra peak ratios and binding energies for LiNiO_2_ and LiNi_0.6_Fe_0.4_O_2_ (Average areas ± standard deviation derived from three independent fitting iterations)

| LiNiO_2_ | Before | Regime 1 | Regime 2 | Regime 3 |
| --- | --- | --- | --- | --- |
| Ni^3+^ Area [%] | 35.3 (±1.2) | 20.0 (±0.3) | 23.1 (±1.3) | 14.9 (±0.5) |
| Ni^2+^ Area [%] | 64.7 (±1.2) | 80.0 (±0.3) | 76.9 (±1.3) | 85.1 (±0.5) |
| Ni^3+^ B.E. [eV] | 857.5 | 857 | 856.7 | 857 |
| Ni^2+^ B.E. [eV] | 856.1 | 855.5 | 855.3 | 855.1 |

| LiNi_0.6_Fe_0.4_O_2_ | Before | Regime 1 | Regime 2 | Regime 3 |
| --- | --- | --- | --- | --- |
| Ni^3+^ Area [%] | 67.1 (±0.3) | 66.4 (±0.1) | 66.3 (±0.3) | 63.0 (±0.1) |
| Ni^2+^ Area [%] | 32.9 (±0.3) | 33.6 (±0.1) | 33.7 (±0.3) | 37.0 (±0.1) |
| Ni^3+^ B.E. [eV] | 856.5 | 856.6 | 856.5 | 856.9 |
| Ni^2+^ B.E. [eV] | 856 | 855.6 | 855.3 | 855.8 |

**Table S5.** Inductively coupled plasma optical emission spectroscopy **(**ICP-OES) measurements of the 1M KOH electrolyte before and after the durability test

| Sample | Li | Ni | Fe |
| --- | --- | --- | --- |
| Pristine KOH | 0.002 | 0.003 | 0.028 |
| KOH after Regime 1 | 0.002 | 0.002 | 0.028 |
| KOH after Regime 2 | 0.002 | 0.003 | 0.029 |
| KOH after Regime 3 | 0.002 | 0.002 | 0.056 |

**Table S6.** Comparison of the oxygen evolution reaction (OER) performance with reported various transition metal oxides

| Sample | Overpotential  @10 mA cm^-2^  [mV] | Tafel slope  [mV dec^-1^] | Electrolyte | Reference |
| --- | --- | --- | --- | --- |
| LiNi_0.6_Fe_0.4_O_2_ | 246 | 41 | 1M KOH | This work |
| LiNiO_2_ | 308 | 90 | 1M KOH | This work |
| LiNiO_2_ | 427 | 82 | 0.1M KOH | ^[2]^ |
| LiNi_0.8_Fe_0.2_O_2_ | 330 | 59 | 0.1M KOH | ^[2]^ |
| LiNiO_2_ | 315 | 57 | 0.1M KOH | ^[3]^ |
| LiNiO_2_ | 270 | 41 | 1M KOH  (with 200 μM Fe^3+^ solution) | ^[3]^ |
| LNO-500 | 300 | 47.2 | 1M KOH | ^[4]^ |
| Li_0.5_Ni_0.5_O_2_ | 370 | 56.6 | 1M KOH | ^[5]^ |
| LiNi_0.9_Fe_0.1_O_2_ | 340 | 46.2 | 1M KOH | ^[5]^ |
| (CoFeNiCrMn)_3_O_4_ | 307 | 30 | 1M KOH | ^[6]^ |
| 1T-MoS2@NPC | 250 | 89 | 1M KOH | ^[7]^ |
| IrO_2_ | 328 | 54 | 1M KOH | ^[3]^ |

**References**

[1]	K. Momma, F. Izumi, *J Appl Crystallogr* **2011**, *44*, 1272.

[2]	K. Zhu, T. Wu, Y. Zhu, X. Li, M. Li, R. Lu, J. Wang, X. Zhu, W. Yang, *ACS Energy Lett.* **2017**, *2*, 1654.

[3]	H. Huang, Y.-C. Chang, Y.-C. Huang, L. Li, A. C. Komarek, L. H. Tjeng, Y. Orikasa, C.-W. Pao, T.-S. Chan, J.-M. Chen, S.-C. Haw, J. Zhou, Y. Wang, H.-J. Lin, C.-T. Chen, C.-L. Dong, C.-Y. Kuo, J.-Q. Wang, Z. Hu, L. Zhang, *Nat Commun* **2023**, *14*, 2112.

[4]	X. Ren, C. Wei, Y. Sun, X. Liu, F. Meng, X. Meng, S. Sun, S. Xi, Y. Du, Z. Bi, G. Shang, A. C. Fisher, L. Gu, Z. J. Xu, *Advanced Materials* **2020**, *32*, 2001292.

[5]	G. Fu, X. Wen, S. Xi, Z. Chen, W. Li, J.-Y. Zhang, A. Tadich, R. Wu, D.-C. Qi, Y. Du, J. Cheng, K. H. L. Zhang, *Chem. Mater.* **2019**, *31*, 419.

[6]	J. Baek, M. D. Hossain, P. Mukherjee, J. Lee, K. T. Winther, J. Leem, Y. Jiang, W. C. Chueh, M. Bajdich, X. Zheng, *Nat Commun* **2023**, *14*, 5936.

[7]	Z. Huang, Y. Bi, J. She, Y. Liu, S. Feng, C. Xu, D. Sun, H. Liu, *Journal of Energy Chemistry* **2025**, *106*, 619.
